# Prognostic Impact of Neuropilin-1 Expression in Egyptian Children with B-lineage Acute Lymphoblastic Leukemia

**DOI:** 10.4084/MJHID.2015.009

**Published:** 2015-01-01

**Authors:** Adel A Hagag, Nahla A Nosair

**Affiliations:** 1 Pediatric Department, Faculty of Medicine, Tanta University, Egypt.; 2 Clinical Pathology Department, Faculty of Medicine, Tanta University, Egypt.

## Abstract

**Background:**

Neuropilins are transmembrane glycoproteins that act as receptors for vascular endothelial growth factors and are involved in the process of tumor angiogenesis.

**Objective:**

The aim of this work was to study the prognostic value of Neuropilin-1 (NRP-1) expression in Egyptian children with B-lineage acute lymphoblastic leukemia (ALL).

**Patients and methods:**

This study was conducted on fifty children with newly diagnosed B-lineage ALL, admitted to Oncology Unit, Pediatric Department, Tanta University Hospitals in the period from August 2010 to March 2014. This series included 32 males and 18 females with ages ranging from 3–17 years and a mean value of 9 ± 3.5 years. Twenty healthy children, age and sex matched, were also included in this study as a control group. For all patients, the following examens were done: Bone marrow aspiration, cytochemistry, immunophenotyping and estimation of Neuropilin-1 expression on blast cells by flow cytometry.

**Results:**

The present study revealed highly significant differences in Neuropilin-1 expression between B-lineage ALL lymphoblasts and control lymphocytes. A significant higher Neuropilin-1 expression was found in pre-B ALL (74.04%) compared with early pre-B (23.55%). Neuropilin-1 positive expression was associated with significantly higher white blood cells count (Mean = 69.3±18.53 ×10^3^/mm^3^ versus 32.5±11.64 ×10^3^/mm^3^ and p=0.003), bone marrow blasts percentage (Mean=76.12±21.4 % versus 41.2±19.71% and p= 0.003), serum lactate dehydrogenase levels (Mean=1992.2 ± 58.6 unit/L versus 955.1± 234.7 unit/L and p=0.001) at diagnosis compared with negative Neuropilin-1 expression. The levels of Neuropilin-1 on BM blasts at diagnosis were higher in patients who subsequently relapsed (Mean=53.8 ± 27.1) or later died (Mean=81.51 ± 9.94) during the period of follow-up compared to those who achieved and maintained complete remission (Mean=18.17 ± 10.4) with p value of 0.001. Furthermore, patients with higher Neuropilin-1 expression had significantly shorter overall survival (Median 27.99 months and p= 0.0133) and disease-free survival (Median=10.23 months and p= 0.0002) than patients with low Neuropilin-1 expression (Median disease-free survival was 38.7 months).

**Conclusion:**

Our findings suggest that Neuropilin-1 is a poor prognosis factor in children with B-lineage ALL and so we recommend the inclusion of Neuropilin-1 as a prognostic marker in children with B-lineage ALL. Its presence at high levels suggests a poor prognosis, and the necessity of intensive therapeutic intervention.

## Introduction

Acute lymphoblastic leukemia (ALL) is the most common childhood malignancy, representing nearly one-third of all pediatric cancers.^1^ With the advent of aggressive multimodality therapy, it has become a curable disease in more than 80% of patients. (2) More than 75% of patients survive free of leukemia recurrence at least 5 years from diagnosis with current treatments that incorporate systemic chemotherapy and specific central nervous system preventive therapy,^3^–^6^ however, the treatment of ALL results in a significant morbidity and mortality.^2^ The use of risk-adapted treatment protocols has improved cure rates while limiting the toxicity of therapy.^7^

Angiogenesis is an important requirement for the development and progression of hematological malignancies including leukemia and lymphoma.^8^ Vascular endothelial growth factor (VEGF) is an important cytokine that contributes to disease evolution in various neoplasms. In particular, VEGF has been described as a mediator of leukemia associated angiogenesis as well as an autocrine growth regulator in leukemic cells.^9^

Neuropilin-1 (NRP-1/BDCA4/CD304) is a transmembrane C-type lectin found on plasmacytoid dendritic cells (pDCs).^10^ It was initially identified as a receptor for class III semaphorins (SEMA3s) mediating neuronal guidance and axonal growth.^11^ It was subsequently found to bind to VEGF that is a critical pro-angiogenic factor that induces proliferation and migration of endothelial cells to tumor vasculature.^12^

Neuropilin-1 expression is reported to be specific for pDCs in humans^13^ and has been found to be highly expressed in diverse solid tumors, as prostate, breast, pancreatic, lung, ovarian and gastrointestinal carcinomas.^14^,^15^ Increased expression of Neuropilin-1 has been correlated with tumor growth and invasiveness.^16^

Furthermore, Neuropilin-1 expression is increased in representative human leukemia and lymphoma cell lines and in a panel of bone marrow specimens obtained from patients with acute lymphoblastic leukemia or acute myeloid leukemia compared with normal bone marrow.^17^ Neuropilin-1 also has been reported to be overexpressed in leukemic lymphocytes in patients with chronic lymphocytic leukemia (CLL).^18^ Therefore, NRP-1 could potentially be used as a target for ligand-directed therapy in leukemia and lymphoma.^17^

## Aim of the Work

The objective of this work was to study the prognostic value of Neuropilin-1 expression in Egyptian children with B-lineage ALL.

## Patients and Methods

The current study was carried out on fifty children after ethical committee approval and written consent of the parents in the Oncology Unit, Pediatric Department, Tanta University Hospitals from August 2010 to March 2014. This series included 32 males and 18 females with an age at diagnosis ranging from 3–17 years and mean value of 9 ± 3.5 years. This study was conducted on fifty patients with newly diagnosed B-lineage ALL attendants to Oncology Unit. Twenty healthy children, age and sex matched, serving as a control group, were also included in the study to estimate the expression of Neuropilin-1 on peripheral blood lymphocyte.

ALL patients were diagnosed on the basis of the clinical presentation, morphological and cytochemical evaluation of blood and marrow smears, together with immunophenotyping. Diagnosis was based on the presence of 20% or more blast cells in bone marrow (BM), according to WHO proposal and the immunophenotyping results consistent with ALL (19) with exclusion of Philadelphia chromosome-positive ALL cases from this study.

The laboratory examinations included: complete blood count, serum LDH levels, bone marrow aspiration, cytochemistry with Sudan black and Myeloperoxidase, immunophenotyping and estimation of Neuropilin-1 expression on blast cells by flow cytometry. The studied patients were treated according to standard protocols for B- lineage ALL^20^ and were monitored during the period of follow-up that lasted for 42 months.

### Flow Cytometry

**Figure 1:**
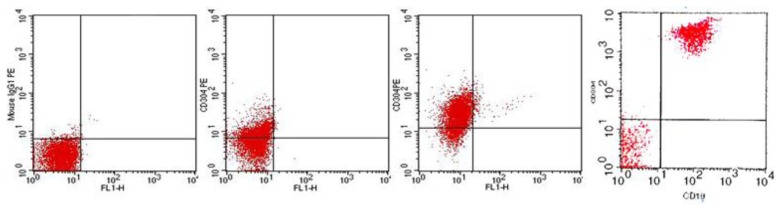
Dot plot showing negative control (left), early pre-B ALL case with positive Neuropilin-1 expression (44.9%), pre-B ALL case with highly positive Neuropilin-1 expression (63.7%) (Middle) and a case of pre-B- ALL with co-expression of Neuropilin-1 and CD19 (right).

Immunophenotyping was performed on gated blast cells from bone marrow samples by flow cytometry using an extensive panel of Fluorescein Isothiocyanate [FITC] and Phycoerythrin [PE] conjugated monoclonal antibodies [MoAbs]. The immunophenotyping of ALL included T-cell lymphoid markers (CD2, CD3, CD5, CD7, CD4, CD8), B-cell markers (CD10, CD19, CD22 and cyto-immunoglobulin) and Myeloid cell markers (CD13, CD33, cyto-MPO).^21^ All MoAbs were purchased from (BD Science, San Jose, CA) while PE-conjugated Neuropilin-1 MoAbs were supplied by (Ancell Corporation, USA). The results of Neuropilin-1 were expressed as a percentage of positively stained cells within the gated blast population. A case was defined as Neuropilin-1 positive if 20% or more of the gated cells expressed it (Figure 1).^17^

### Statistical analysis

Data were analyzed using SPSS version 20. Quantitative data were expressed in the form of mean and standard deviation while qualitative data were described in the form of number and percentage. Differences between groups were evaluated with student t-test for quantitative data and Chi-square test and ANOVA for qualitative data. The statistics and survival analysis were carried out according to Kaplan-Meier product limit estimates.

## Results

There were 31 patients (62% of total) with positive NRP-1 expression (Neuropilin- present in ≥ 20% of blast population) and 19 patients (38% of total) with negative Neuropilin-1 expression (< 20% of BM blasts expressing Neuropilin-1) (Table 1).

There were no statistically significant differences between Neuropilin-1 positive and Neuropilin-1 negative patients regarding age, sex, lymphadenopathy, hepatosplenomegaly, CNS involvement, hemoglobin levels or platelets count, while there were statistically significant differences between Neuropilin-1 positive and Neuropilin-1 negative expression regarding leukocytes count, percentage of BM blast cells and serum LDH levels with higher leucocytes count, percentage of BM blasts and serum LDH in Neuropilin-1 positive patients (Table 2).

The studied patients were categorized on the basis of immunophenotyping into early pre-B ALL (32 cases, 64%) and pre-B ALL (18 cases, 36%). Neuropilin-1 was significantly higher in pre-B compared with early pre-B ALL patients (Table 3).

Thirty patients (60%) achieved and maintained complete remission (CR) till the end of the study; 12 patients (24%) suffered from relapse and eight patients died either during induction or maintenance therapy. A greater number of patients with complete remission were Neuropilin-1 negative than Neuropilin-1 positive (17/30). Most of ALL patients who relapsed, were Neuropilin-1 positive (10/12) with a mean expression percentage of 53.8±27.12 and all patients who died were positive for Neuropilin-1 at diagnosis (8 cases). Neuropilin-1 expression was significantly higher in relapsed patients and in patients who died during therapy when compared to patients who achieved complete remission (Table 4).

**Figure 2:**
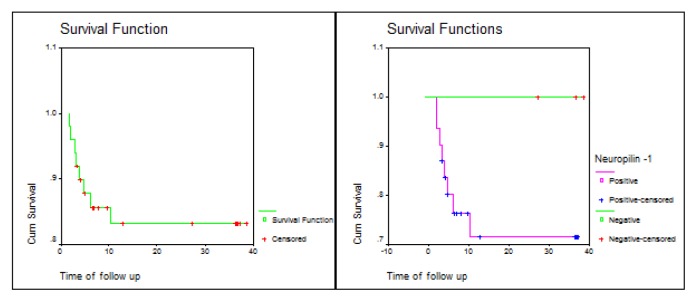
Overall survival (OS) of Neuropilin-1 positive and negative ALL patients.

**Figure 3:**
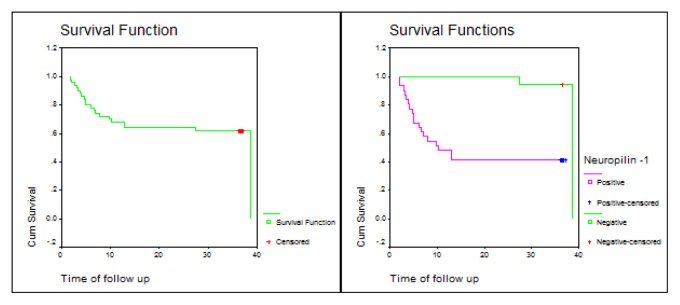
Disease-free survival (DFS) of Neuropilin-1 positive and negative ALL patients.

There was a statistically significant difference in prognosis between Neuropilin-1 positive and negative patients, with a significantly shorter overall survival (OS) and disease-free survival (DFS) in Neuropilin-1 positive B-lineage ALL patients (Table 5 and Figure 2 and 3).

## Discussion

Acute lymphoblastic leukemia (ALL) is the most common childhood malignancy, representing nearly one-third of all pediatric cancers.^1^ With the advent of aggressive multimodality therapy, it has become a curable disease in over than 80% of patients, however, the treatment of ALL results in a significant morbidity and mortality.^2^ The use of risk-adapted treatment protocols has improved cure rates while limiting the toxicity of therapy.^7^

The present study measured Neuropilin-1 surface expression on BM blasts in 50 children with newly diagnosed B-cell ALL compared to normal peripheral blood lymphocytes from 20 healthy controls by flow cytometry.

Neuropilin-1 was expressed in all patients with B-lineage ALL included in this study with variable degrees of expression ranging from 7.9% to 92.1% of BM blasts. 31 patients presented high Neuropilin-1 expression ≥ 20% ( range: 20.5–92.1) (Neuropilin-1 positive group) and 19 patients low expression, less than 20% of BM blasts ( range: 7.9–14.5) (Neuropilin-1 negative group), while expression in peripheral blood normal lymphocytes did not exceed 3.4%. A highly significant statistical difference in levels of Neuropilin-1 expression was found between ALL patients and controls and between positive and negative Neuropilin-1 expression groups of patients.

This is in agreement with Karjalainen et al 2011^17^ who examined Neuropilin-1 in patients with acute leukemia and demonstrated its expression, above baseline bone marrow levels, in all B-cell ALL samples and in two thirds of AML samples with stronger expression in blast cells of B-cell ALL than AML blast cells.^17^ Similarly, Meyerson et al 2012^22^ found that Neuropilin-1 is frequently expressed on B-ALL blasts (71%), whereas its expression is less frequent on AML blasts (22.9%) and consistently absent on peripheral blood lymphocytes.^22^

The present study revealed that the mean percentage of expression of Neuropilin-1 in bone marrow blasts in B-lineage ALL patients was 36.86% overall. Neuropilin-1 expression was significantly higher in patients with pre-B acute lymphoblastic leukemia (74.04%) than patients with early pre-B ALL (23.55%).

This datum is in agreement with Meyerson et al 2012^22^ who found that Neuropilin-1 is frequently expressed on B-ALL blasts, and weakly expressed in normal bone marrow B-cell progenitors, while gradually decreasing during maturation, to be completely lost at later stages of B-cell. The expression of Neuropilin-1 on B-cell progenitors may explain its frequent higher expression in precursor B-ALL than mature ALL**.**^22^

In our study, Neuropilin-1 expression was significantly associated with higher white blood cells Count, BM blasts percentage, and serum LDH levels at diagnosis. There were significantly higher levels of Neuropilin-1 expression on bone marrow blasts at diagnosis in patients who subsequently relapsed or died during the period of follow-up compared to those who achieved and maintained complete remission. Also, patients with higher Neuropilin-1 expression had significantly shorter overall survival and disease-free survival than patients with low Neuropilin-1 expression. These results indicate that higher Neuropilin-1 expression levels correlated with disease severity and biologic progression in children with B-Lineage ALL. This datum is in agreement with previous studies establishing the poor prognostic impact of Neuropilin-1 expression also on AML.^9^,^23^,^24^,^25^

Importance of Neuropilin-1, as marker of disease in pediatric acute lymphoblastic leukemia, is further stressed by Beesley et al 2005^26^ who identified Neuropilin-1 as a part of gene expression signature associated with relapse and adverse clinical outcome, and by Solly et al. 2012^27^ who considered Neuropilin-1 an useful marker of minimal residual disease.

## Conclusions

Our findings suggest that Neuropilin-1 expression on bone marrow blasts is a valuable marker of bad prognosis in patients with B-lineage ALL. Therefore, we recommend the incorporation of Neuropilin-1 expression on bone marrow blasts in children with B-cell ALL as a prognostic marker, useful to categorize patients into the bad prognosis group and then candidate for an intensive treatment.

## Figures and Tables

**Table 1:** Comparison between Neuropilin-1 positive and negative groups of patients regarding Neuropilin-1 expression.

	Neuropilin-1 positive group (n=31)	Neuropilin-1 negative group (n=19)
Range	20.5–92.1	7.9–14.5
Mean ± SD (Mean percentage)	52.87±13.33	10.73±2.11
t. test	48.071	
p. value	0.001*	

* Significant P value <0.05

**Table 2:** Comparison between Neuropilin-1 positive and negative groups of patients regarding clinical and laboratory data.

Parameters		Neuropilin-1 positive (n=31)		Neuropilin-1 negative (n=19)		t test or X^2^	P value
		N	%	N	%		
**Sex**	Males	19	61.29	13	68.42	0.260	0.610
	Females	12	38.71	6	31.58		
**Age (years)**	Range Mean ± SD	2–10 6.74±3.23		2–10 7.55±2.63		0.536	0.336
**Lymphadenopathy**	+ve	12	37.71	9	47.37	0.361	0.547
	−ve	19	61.29	10	52.63		
**HSM**	+ve	20	64.52	13	68.42	0.081	0.777
	−ve	11	35.48	6	31.58		
**CNS infiltration**	+ve	3	9.68	1	5.26	0.311	0.576
	−ve	28	90.32	18	94.74		
**Hb (gm/dl)**	Range Mean ±SD	5.6–11.2 8.10±1.23		6–10 7.69±1.95		0.947	0.584
**Platelets (x10^3^/mm^3^)**	Range Mean ±SD	20.9–84.3 42.15±17.48		27.6–98.2 49.10±19.37		0.635	0.447
**TLC (x10^3^/mm^3^)**	Range Mean ±SD	21.5–178.1 69.30±18.35		24.4–61.2 32.50±11.64		10.325	0.001*
**BM blast (%)**	Range Mean ±SD	55.1–98.6 76.12±21.40		32.5–62.3 41.20±19.71		9.325	0.008*
**LDH (U/L)**	Range Mean ±SD	1950–2020 1992.2±581.6		790.1–1240 955.1±234.7		15.417	0.001*

* Significant P value <0.05.

HSM = Hepatosplenomegaly, CNS = central nervous system, TLC = Total leucocytes count, BM = Bone marrow, LDH = Lactate dehydrogenase. BM blasts % = Mean percentage of BM blasts.

**Table 3:** Neuropilin-1 expression in different immunological B- lineage ALL subtypes.

	Neuropilin-1 expression			
	Early pre-B (n=32)		Pre-B (n=18)	
	CD10 positive (n=32)	CD10 negative (n=0)	CD10 positive (n=6)	CD10 negative (n=12)
**Range**	8.6–57.2		15.5–94.3	
**Mean ±SD**	24.51±12.16		75.12±21.3	
**ANOVA test**	25.33			
**p. value**	0.001*			

* Significant P value <0.05.

Mean = mean percentage.

**Table 4:** Neuropilin-1 expression in relation to outcome of ALL.

	Neuropilin-1 expression		
	Complete remission (no=30)	Relapse (no=12)	Death (no=8)
**Range**	7.9–46.1	12.9–91.2	61.9–92.1
**Mean ±SD**	18.17±10.4	53.8±27.12	81.51±9.94
**ANOVA test**	33.628		
**p. value**	0.001*		

* Significant P value <0.05

**Table 5:** Log Rank test of overall and disease-free survival.

	Overall survival			Log Rank	
	Median (Months)	SE	CI 95%	test value	P-value
**All**	32.96	1.86	(29.33, 36.60)		
**Negative**	-	-	-	6.13	0.0133*
**Positive**	27.99	2.79	(22.52, 33.46)		
	**Disease free survival**			**Log Rank**	
	Median (Months)	SE	CI 95%	test value	P-value
**All**	38.7	2.28	(22.25, 31.19)		
**Negative**	38.7	0.82	(36.50, 39.71)	13.63	0.0002*
**Positive**	10.23	3.32	(3.73, 16.74)		

* Significant
